# Tinting Vegetable Tissues

**Published:** 1869-04

**Authors:** 


Tinting Vegetabe Tissues.—At a meeting of the Botanical Society of Edinburgh, Dr. W. R. McNab described the results of his recent attempts at staining tissues with various dyes. He mentioned a large series of experiments he had made by staining certain microscopical structures with acetate of mauvine and Beale's carmine solution. He showed that by means of staining, the high powers of the microscope can be used to bring out points of structure not easily demonstrated without being so treated. The process of staining does not seem to be attended with any great difficulty, and the author believes that very important results may be obtained by careful study of its action on germinating plants.

